# Correlation between allergic diseases and lung cancer: a systematic review and meta-analysis

**DOI:** 10.3389/fmed.2025.1560000

**Published:** 2025-07-16

**Authors:** Kunpeng Yang, Hui Zhao, Lei Wang, Chenglun Cai, Peiyun Lv, Bao Wang

**Affiliations:** ^1^Jilin Cancer Hospital, Changchun, China; ^2^Changchun University of Chinese Medicine, Changchun, China; ^3^Tianjin Medical University, Tianjin, China

**Keywords:** allergic rhinitis, eczema, lung cancer, meta-analysis, systematic review

## Abstract

**Objectives:**

This meta-analysis aims to investigate the potential association between allergic diseases and lung cancer.

**Methods:**

A systematic search was conducted in PubMed, Cochrane Library, Embase, and Web of Science databases up to October 8, 2024. Data analysis was performed using Stata 14.0, employing fixed or random effects models based on heterogeneity (*p* > 0.1, I^2^ ≤ 50% or I^2^ > 50%). Subgroup and sensitivity analyses were conducted, and publication bias was assessed.

**Results:**

Analysis of 10 studies revealed a negative association between allergic diseases and lung cancer risk (OR: 0.75, 95% CI: 0.66–0.85, I^2^ = 74.4%, *p* < 0.001). Subgroup analyses showed eczema was not statistically associated with lung cancer (OR = 0.73; 95% CI: 0.51–1.06), while allergic rhinitis showed negative correlation (OR = 0.74; 95% CI: 0.64–0.86). Both men (OR = 0.56; 95% CI: 0.44–0.71) and women (OR = 0.71; 95% CI: 0.54–0.94) with allergic diseases demonstrated reduced lung cancer risk.

**Conclusion:**

Allergic diseases are inversely associated with lung cancer risk, with allergic rhinitis acting as a protective factor, while eczema shows no significant association. Further epidemiological studies are warranted.

**Systematic review registration:**

https://inplasy.com/inplasy-2024-11-0086/, identifier INPLASY2024110086.

## Introduction

Allergies and cancer are increasingly recognized as significant health issues in both developed and developing countries. In developed nations, nearly one in five individuals suffer from at least one allergy, while the prevalence in developing countries is even higher (1). Lung cancer, widely regarded as one of the most prevalent cancer types worldwide, poses a significant public health concern. According to the latest research estimates, there were approximately 2.26 million new cases of lung cancer reported globally in 2019, leading to around 2.04 million deaths. From 2010 to 2019, the number of new lung cancer cases increased by 26.3%, while the death rate rose by 20.9%. Furthermore, the disability-adjusted life years (DALYs) attributable to lung cancer rose by 16.0%, making it the second leading cause of cancer-related mortality and DALYs globally, after cardiovascular diseases (2).

The association between allergic diseases and the risk of lung cancer varies based on the specific type of allergy. Most studies suggest that a history of asthma is linked to an increased risk of lung cancer, whereas a history of eczema or allergic rhinitis (AR) may decrease the risk. This contradictory finding provides evidence for two distinct hypotheses (3). Studies indicate that the global prevalence of allergic rhinitis ranges from 5 to 50%, with epidemiological data demonstrating a consistent upward trend over time (4). Allergic conditions, including AR and eczema, occur due to the interaction between environmental allergens and immunoglobulin E (IgE) on mast cells found in skin or mucosal tissues. This interaction triggers the release of mediators, such as histamine, leukotrienes, and cytokines (5), which initiate localized inflammatory responses that can spread throughout the body (6). These conditions are associated with various health issues, including depression and sleep disturbances, and also lead to reduced productivity and significant healthcare costs (7).

However, despite the heightened attention on the relationship between allergic diseases and lung cancer, considerable controversy and uncertainty persist regarding the specific associations between eczema, AR, and lung cancer. This may arise from limitations such as limited sample sizes, inadequate adjustment for confounding variables, and inherent selection bias within study methodologies (8). Based on mechanistic evidence of IgE-mediated immune surveillance, we hypothesize that AR and eczema reduce lung cancer risk via enhanced antitumor immunity, with effects modulated by allergy type, gender, and geographic context. To test this hypothesis, we conducted a meta-analysis to evaluate the association between AR/eczema and lung cancer risk. By integrating results across populations, we aim to clarify whether allergic diseases serve as protective factors and to identify potential biological mechanisms. This approach not only helps clarify the potential role of allergic diseases in the development of lung cancer but also provides new insights and evidence for risk assessment, early detection, and the formulation of personalized treatment strategies for lung cancer.

## Methods

This study adhered to the guidelines of the Preferred Reporting Items for Systematic Reviews and Meta-Analyses (PRISMA) (9) and was registered on the International Platform of Registered Systematic Review and Meta-analysis Protocols (INPLASY) platform (INPLASY2024110086).

### Data sources and searches

We conducted a systematic search of databases, including PubMed, Cochrane Library, EMBASE, and Web of Science, spanning the period from the inception of each database to October 8, 2024, without language restrictions. The search employed medical subject headings (MeSH) and keywords such as “eczema,” “allergic rhinitis,” “hay fever,” “lung cancer,” and “lung tumor.” Furthermore, we manually examined the references of included studies, as well as other published systematic reviews and relevant bibliographies, to identify further pertinent research. A detailed description of the search strategy is provided in Supplementary material 1.

### Eligibility criteria

The inclusion criteria for this study were as follows: (1) cohort studies or case–control studies; (2) investigations exploring the association between allergic diseases such as eczema and AR and the risk of lung cancer, with lung cancer risk considered as the outcome; and (3) providing adjusted odds ratios (OR) along with their 95% confidence intervals (CI). When a study presents multiple results, it is recommended to prioritize those that are associated with the longest follow-up time or the largest number of participants. The exclusion criteria included conference abstracts, study protocols, duplicate publications, and studies lacking relevant outcomes.

### Study selection

Two reviewers (ZH and YKP) independently conducted a literature screening based on predetermined eligibility and exclusion criteria. Initially, they excluded duplicate and irrelevant articles by reviewing the titles and abstracts. Subsequently, they downloaded and thoroughly evaluated the full texts of potentially eligible studies to identify all that met the inclusion criteria. Any discrepancies in the screening process were resolved by a third reviewer (WB), who served as the final decision-maker.

### Data extraction

Two independent reviewers (ZH and YKP) utilized standardized data collection forms to extract information and cross-checked it for accuracy. They documented key data from each included study, including the first author, publication year, study country or region, number of participants, study design, pertinent age information, as well as the results and their corresponding 95%CI. Additionally, they recorded details concerning the type of allergy disease, lung cancer diagnosis, and the adjusted confounding factors. In the event of any discrepancies, the ultimate decision was made by the third reviewer, WB.

### Risk of bias assessment

This study employed the Newcastle-Ottawa Scale (NOS) to evaluate the quality of the included cohort and case–control studies (10). The NOS assesses studies across three domains: selection, comparability, and outcomes (for cohort studies) or exposure (for case–control studies). Based on NOS scores, we classified study quality into three categories: low quality (0–3 points), moderate quality (4–6 points), and high quality (7–9 points). Each study’s quality was independently evaluated by two reviewers, with any discrepancies resolved by consensus or through discussion.

### Statistical analysis

This study used Stata software version 14.0 to extract adjusted OR and their corresponding 95% CI from the included studies, to evaluate the association between allergic diseases and lung cancer risk. Heterogeneity was assessed using the chi-square test and I^2^ statistic; a fixed-effects model was applied when *p* > 0.1 and I^2^ ≤ 50%, while a random-effects model was employed when I^2^ > 50%. Sensitivity analyses were performed to ascertain the robustness of the findings, and publication bias was examined through visual inspection of funnel plots as well as Egger’s regression test. Furthermore, subgroup analyses were conducted based on allergy type, gender, and geographic region.

## Results

### Search results

**Figure 1:**
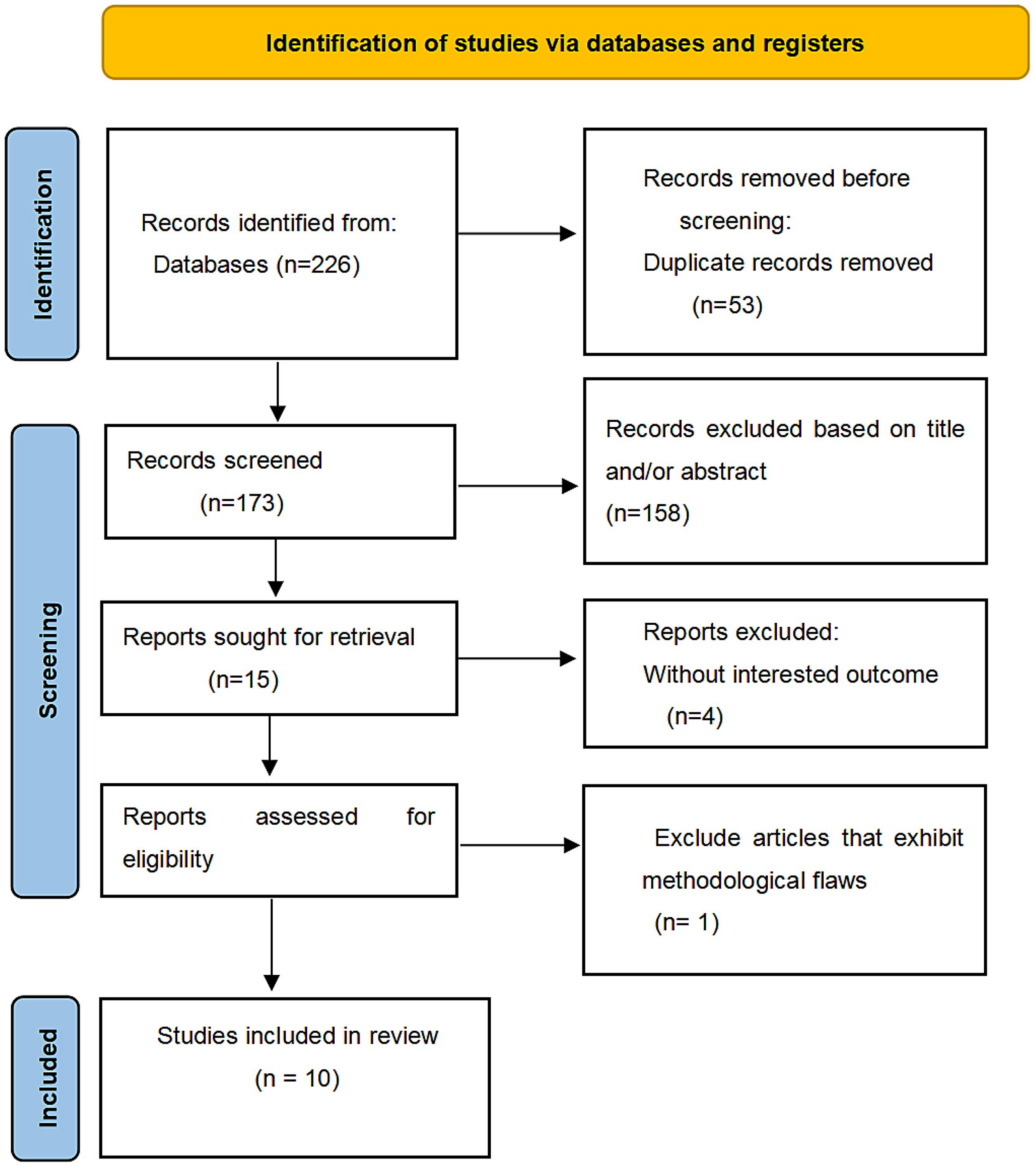
The complete process of eligible literature screening.

A total of 226 articles were retrieved from the systematic search conducted up to October 8, 2024. First, 53 duplicate articles were excluded, followed by a screening process that excluded an additional four articles due to their titles and abstracts. Subsequently, after a comprehensive review of all the articles, we eliminated one article due to significant controversy and the absence of crucial variables (11). Ultimately, this study includes a total of ten studies (3, 12–20), as shown in Figure 1.

### Study characteristics

This meta-analysis included 10 studies encompassing a total of 3,870,795 participants over a time span from 2000 to 2019. There were 2 cohort studies (19, 20) and 8 case–control studies (3, 12–18), with sample sizes ranging from 302 to 1,744,575. The diagnosis of eczema and AR was established using questionnaires or by measuring serum IgE levels, while lung cancer diagnosis was determined through pathological histology or using International Classification of Diseases codes 9 or 10 (ICD-9 or 10). All studies reported adjusted OR, albeit with slight variations in the confounding factors adjusted for across studies. The key characteristics of these studies are summarized in Table 1.

**Table 1:** Basic characteristics of the included studies.

Author	Year	Country	Study type	Sample size	Age (years)	Study period	Diagnosis of allergic disease	Diagnosis of lung cancer
Monica E. D’Arcy	2019	America	Case–control	Total: 2744575 Case group: 1744575 Control group: 1000000	66–99	1992–2013	ICD-9	ICD-9
Mariam El-Zein	2014	Canada	Case–control	Total: 2655 Case group: 1169 Control group: 1486	35–75	1996–2002	Questionnaires	Histologically
Mariam El-Zein	2010	Canada	Case–control	Total: 3821 Case group: 512 Control group: 3300	35–70	1979–1985	Questionnaires	Histologically
Marine Castaing	2005	European	Case–control	Total: 5970 Case group: 2854 Control group: 3116	28–82	1998–2001	Questionnaires	Histologically
Hao Wang	2006	Germany	Case–control	Total: 4467 Case group: 196 Control group: 4271	50–74	2000–2002	Questionnaires IgE diagnosis	Histologically
Olga Y. Gorlova	2006	America	Case–control	Total: 522 Case group: 280 Control group: 242	Case: 60.2 ± 12.7 Control: 61.9 ± 10.9	1995–2003	Questionnaires	Histologically
Kathryn E. Osann	2000	America	Case–control	Total: 302 Case group: 98 Control group: 204	Case: 61.7 Control: 62.6	1990–1993	Questionnaires	Histologically
Matthew B. Schabath	2005	America	Case–control	Total: 2928 Case group: 1375 Control group: 1553	Cases: 61.8 ± 10.7 Control: 61.2 ± 9.7	1995–2003	Questionnaires	Histologically
Alison Talbot-Smith	2003	Australia	cohort study	Total: 3308 Male: 1522 Female: 1786	Male: 50.1 ± 16.7 Female: 50 ± 16.6	1981–1999	Questionnaires	Histologically ICD-10
Michelle C. Turner	2005	America	cohort study	Total: 1102247 Male: 483080 Female: 619167	≥45	1982–2000	Questionnaires	ICD-9 ICD-10

Author	Allergic disease type	Confounders adjusted	Adjusted OR	NOS scores
Monica E. D‘Arcy	Eczema Allergic rhinitis	Sex, age, race, smoking	Eczema: 1.03 (0.85–1.24) Allergic rhinitis: 0.79 (0.74–0.85)	7
Mariam El-Zein	Eczema Allergic rhinitis	Sex, age, race, education, fruit and vegetable consumption, smoking	Eczema: 0.78 (0.47–1.28) Allergic rhinitis: 0.38 (0.23–0.63)	7
Mariam El-Zein	Eczema	Ancestry, smoking, occupational exposure to asbestos and silica	Eczema: 0.29 (0.1–0.7)	7
Marine Castaing	Eczema	Age, sex, study center, cumulative tobacco, smoking	Eczema: 0.61 (0.48–0.76) Male: 0.6 (0.45–0.81) Female: 0.62 (0.42–0.92)	6
Hao Wang	Eczema Allergic rhinitis	Age, education, BMI, family history of cancer, smoking, alcohol	Eczema: 1 (0.38–2.57) Allergic rhinitis: 0.34 (0.11–1.1)	7
Olga Y. Gorlova	Allergic rhinitis	Age, gender, race, education and income	Allergic rhinitis: 0.5 (0.3–0.83) Male: 0.44 (0.16–1.2) Female: 0.51 (0.27–0.96)	6
Kathryn E. Osann	Allergic rhinitis	Age, education, and smoking.	Allergic rhinitis: 1.3 (0.5–3.6)	7
Matthew B. Schabath	Allergic rhinitis	Age, gender, ethnicity, smoking, pack-years of cigarette smoking, exposure to asbestos and wood dust	Allergic rhinitis: 0.58 (0.48–0.7)	6
Alison Talbot-Smith	Allergic rhinitis	Age, smoking	Male: 0.64 (0.08–4.87) Female: 1.45 (0.4–5.3)	7
Michelle C. Turner	Allergic rhinitis	Gender, race, smoking, BMI, education, family history of non-Hodgkin’s lymphoma.	Allergic rhinitis with smoking: 0.85 (0.8–0.9) Allergic rhinitis without smoking: 1.02 (0.86–1.21)	7

### Risk of bias assessment

We utilized the NOS to assess the methodological quality of the 10 studies, of which seven (3, 12, 13, 15, 17, 19, 20) were rated as high quality and three (14, 16, 18) as moderate quality. The mean NOS score was 6.7, indicating a generally high methodological standard among the studies included in this meta-analysis. Detailed scores are presented in Table 1.

### Allergic diseases and risk of lung cancer

**Figure 2:**
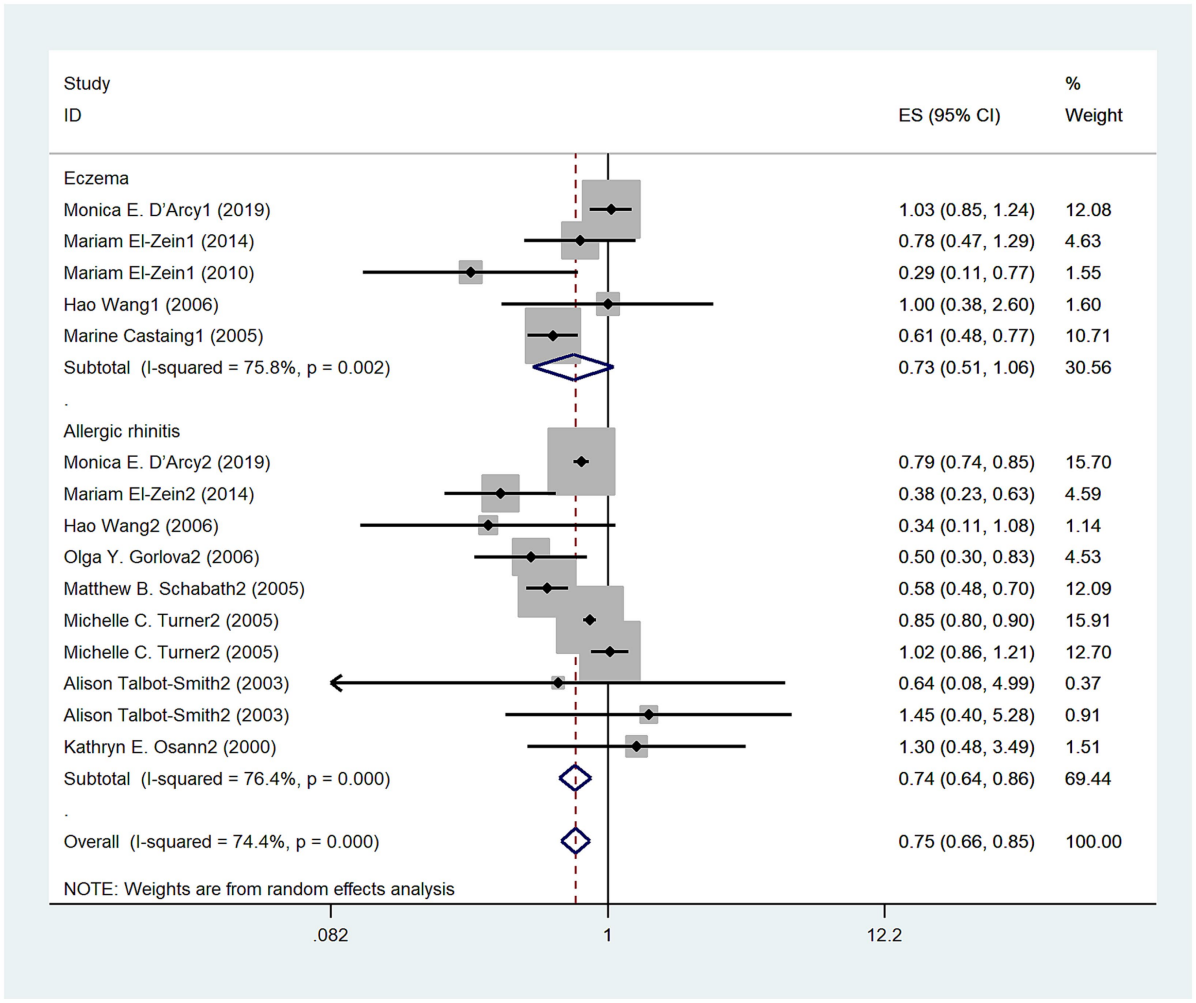
Meta-analysis of the association between eczema, allergic rhinitis (AR), and lung cancer risk.

A total of ten studies were conducted to examine the relationship between allergic diseases and the risk of lung cancer. The meta-analysis revealed a negative correlation between allergic diseases and lung cancer risk (OR: 0.75, 95% CI: 0.66–0.85, I^2^ = 74.4%, *p* < 0.001; Figure 2). Despite the observed significant heterogeneity, sensitivity analyses showed that none of the individual studies significantly influenced the overall effect size, thus confirming the robustness of our findings (Supplementary Figure A).

### Type of allergy and risk of lung cancer

Five studies (3, 12–15) explored the relationship between eczema and the risk of lung cancer. Overall, no significant association was observed between eczema and lung cancer risk (OR = 0.73, 95% CI: 0.51–1.06; I^2^ = 75.8%, *p* = 0.002; Figure 2). In contrast, seven studies (3, 12, 16–20) investigated the relationship between AR and lung cancer risk, demonstrating a negative correlation (OR = 0.74, 95% CI: 0.64–0.86; I^2^ = 76.4%, *p* < 0.001; Figure 2).

### Gender and risk of lung cancer

**Figure 3:**
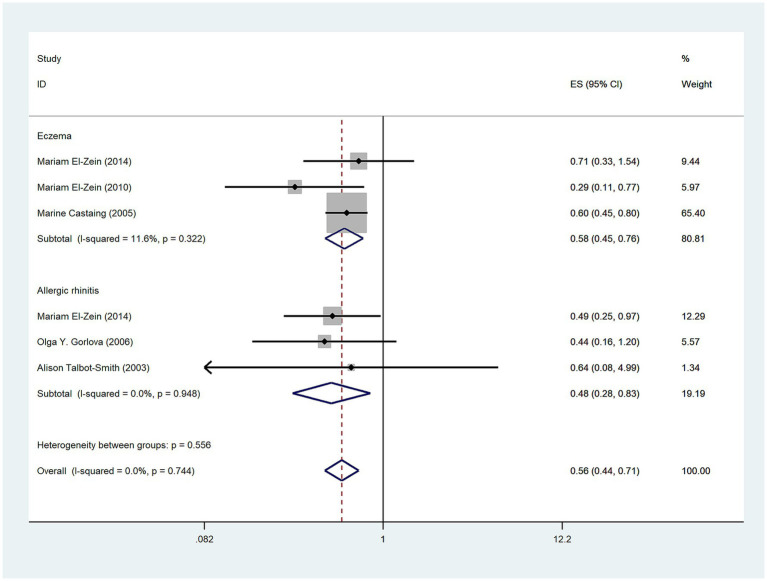
Meta-analysis of allergic diseases and lung cancer risk in men. Relationship between allergic diseases, eczema, and allergic rhinitis (AR) with lung cancer risk in men.

**Figure 4:**
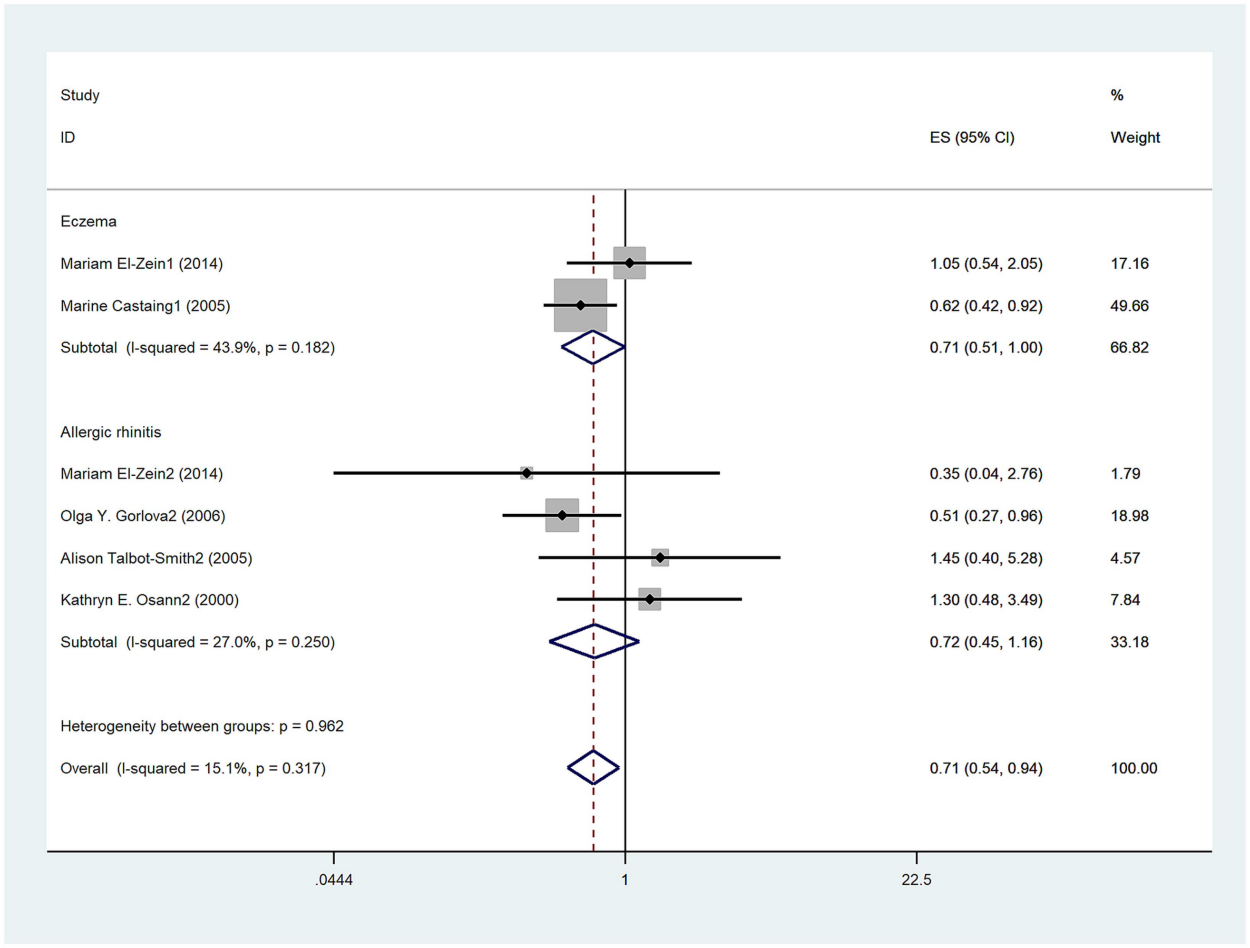
Meta-analysis of allergic diseases and lung cancer risk in women. Relationship between allergic diseases and allergic rhinitis (AR) with lung cancer risk in women.

The aim of five studies (3, 13, 14, 16, 19) was to assess the relationship between allergic diseases and lung cancer risk in men. The results suggested a negative association between allergic diseases and lung cancer risk in men (OR = 0.56, 95%CI: 0.44–0.71; I^2^ = 0%, *p* = 0.744; Figure 3). Three studies (3, 13, 14) examined the relationship between eczema and lung cancer risk in men, revealing a negative correlation (OR = 0.58, 95%CI:0.45–0.76; I^2^ = 11.6%, *p* = 0.322; Figure 3). Furthermore, three studies (3, 16, 19) investigated the relationship between AR and lung cancer risk in men, demonstrating a persistent negative correlation (OR: 0.48, 95% CI: 0.28–0.83; I^2^ = 0%, *p* = 0.948; Figure 3). Five studies (3, 14, 16, 17, 19) explored the relationship between allergic diseases and lung cancer risk in women, showing a negative correlation (OR = 0.71, 95% CI: 0.54–0.94; I^2^ = 15.1%, *p* = 0.317; Figure 4). Four studies (3, 16, 17, 19) investigated the relationship between AR and lung cancer in women, finding no statistically significant association (OR = 0.72, 95% CI: 0.45–1.16; I^2^ = 27%; *p* = 0.25; Figure 4).

### Types of territory and risk of lung cancer

**Figure 5:**
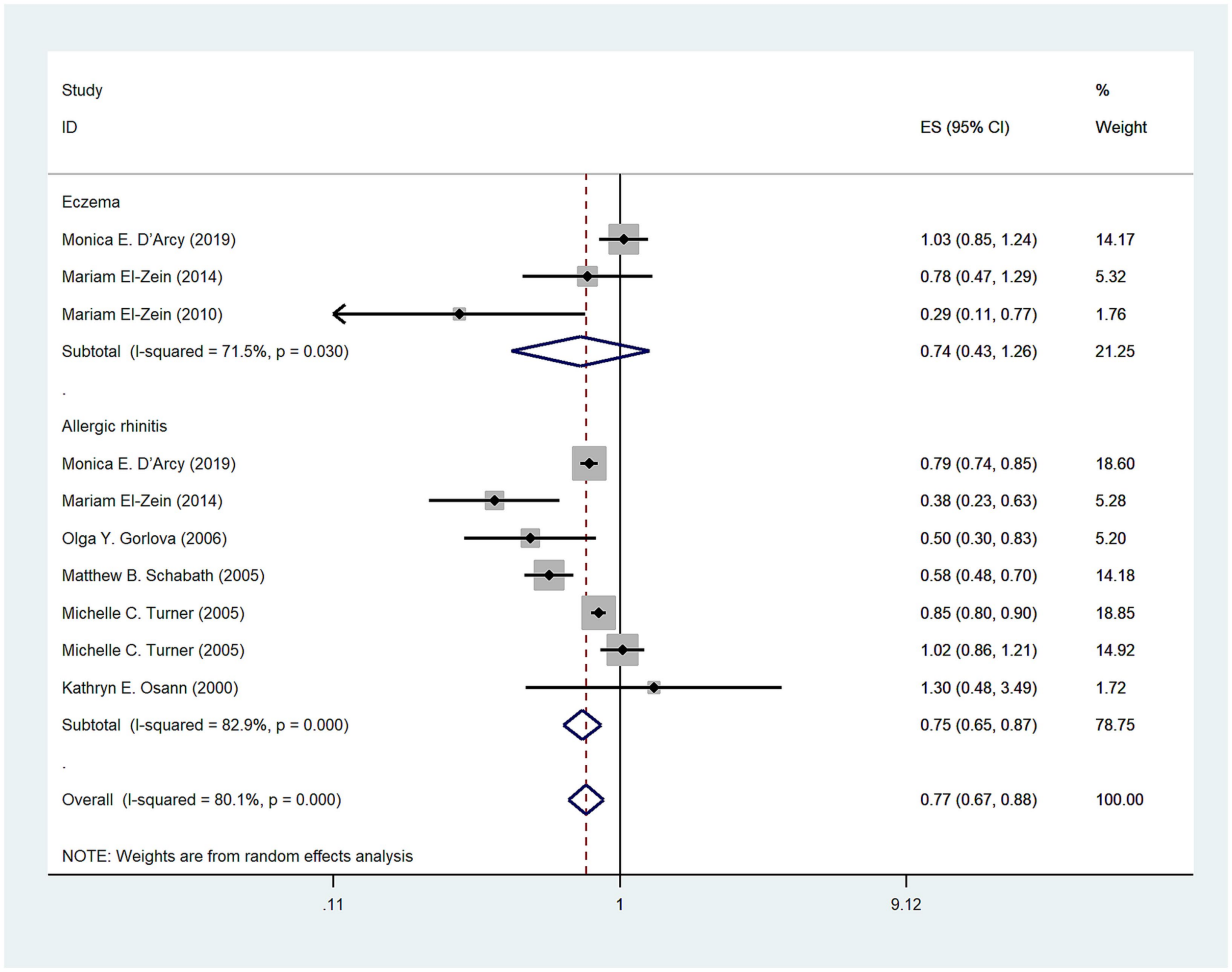
Meta-analysis of allergic diseases and lung cancer risk in America. Relationship between allergic diseases, eczema, and allergic rhinitis (AR) with lung cancer risk in America.

A total of seven studies (3, 12, 13, 16–18, 20) investigated the relationship between allergic diseases and lung cancer risk on the American continents and revealed a statistically significant negative association (OR = 0.77, 95% CI: 0.67–0.88; I^2^ = 80.1%, *p* < 0.001; Figure 5). Three studies (3, 12, 13) investigated no association between eczema and lung cancer on the American continents (OR = 0.74, 95% CI: 0.43–1.26; I^2^ = 71.5%, *p* = 0.03; Figure 5). Additionally, six studies (3, 12, 15–18, 20) investigated a negative association between AR and lung cancer risk on the American continents (OR = 0.75, 95% CI: 0.65–0.87; I^2^ = 82.9%, *p* < 0.001; Figure 5).

### Publication bias

**Figure 6:**
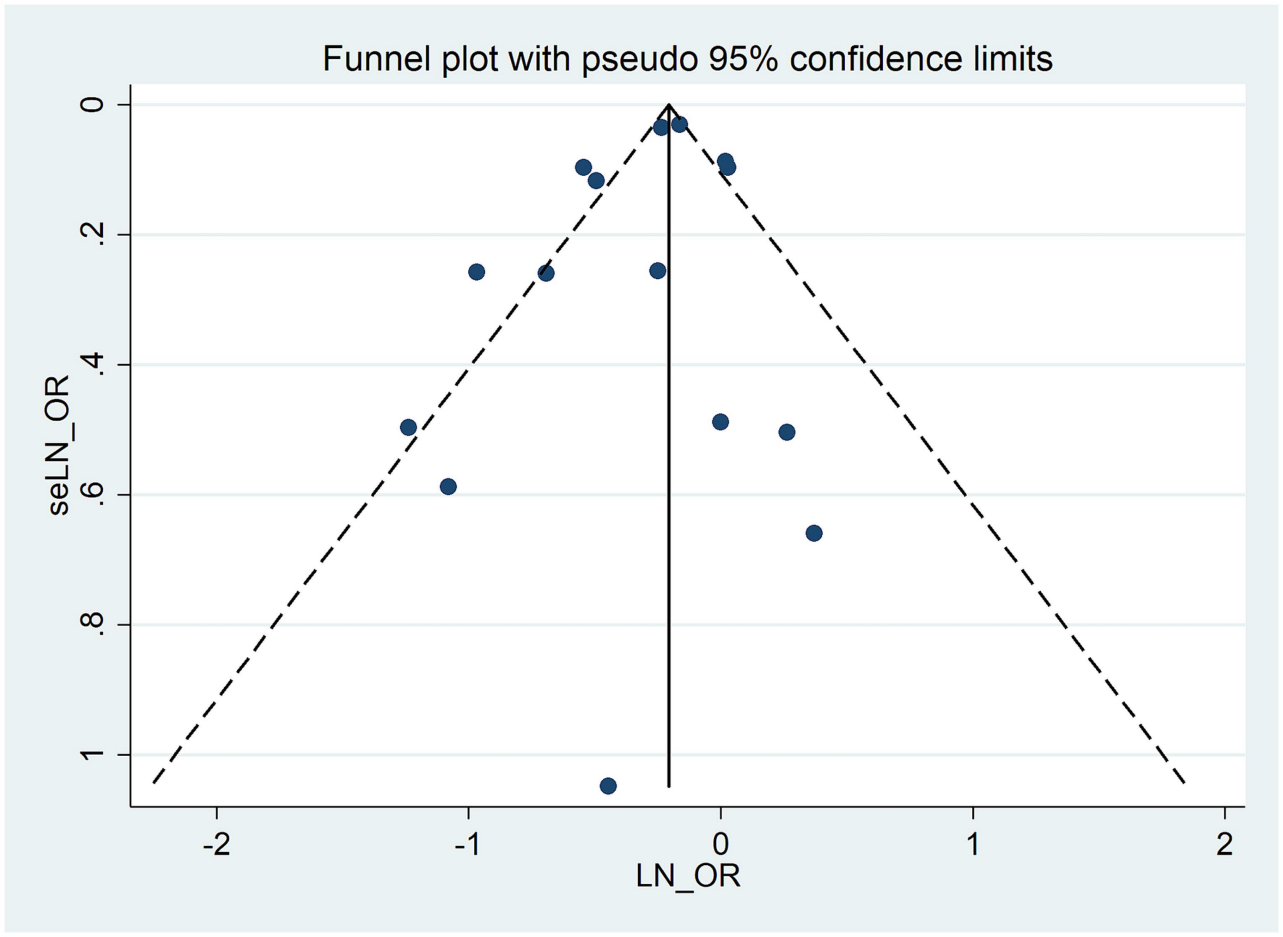
Funnel plot for publication bias in the meta-analysis of allergic diseases and lung cancer risk.

A visual assessment of the funnel plot combined with the Egger regression test (*p* = 0.247) did not provide significant evidence for publication bias (Figure 6), thus suggesting that there is no evidence of such bias in the meta-analysis investigating the association between allergic diseases and lung cancer risk.

## Discussion

This meta-analysis encompasses 10 cohort studies involving a total of 3,870,795 participants, offering a comprehensive evaluation of the association between allergic diseases and lung cancer. Our findings indicate that individuals with allergies exhibit a 0.25-fold reduction in the risk of developing lung cancer compared to the control group without allergic conditions, with a negative correlation observed in both male and female subgroups. AR demonstrates a negative association with lung cancer risk, particularly significant among males and in the Americas; however, no statistically significant correlation was identified between AR and lung cancer in females. Additionally, there is no statistically significant relationship between eczema and lung cancer risk overall, although a negative association was noted within the male subgroup. Previous studies have demonstrated that allergic diseases may reduce the risk of lung cancer by enhancing immune surveillance, thus indicating that individuals with a history of allergies exhibit a lower likelihood of developing lung cancer (8). Our study corroborates this conclusion. We incorporated additional relevant and recently published research into our analysis, which further investigates the relationships between various allergic diseases, gender, study region, and lung cancer risk.

While asthma is an allergic condition, it was intentionally excluded from this meta-analysis due to its established association with increased lung cancer risk in epidemiological literature (21), which contrasts with the protective associations observed for allergic rhinitis and eczema. This exclusion ensured methodological homogeneity in evaluating allergic diseases with hypothesized protective effects against lung carcinogenesis and avoided conflating distinct immunological pathways. This approach ensures robust, interpretable findings on AR/eczema as potential protective factors for lung cancer.

Although epidemiological studies have provided evidence suggesting a link between allergic diseases and cancer risk, the precise nature of this relationship remains contentious (19), with two primary hypotheses proposed to explain the association between allergy history and cancer risk. The immune surveillance hypothesis proposes that allergies may decrease cancer risk, as IgE-mediated allergic reactions may indicate an heightened state of immune surveillance marked by interleukin and eosinophil activity. This enhanced immune response could aid in the elimination of malignant cells in the early stages of tumorigenesis (22). Conversely, the antigen hypothesis posits that chronic immune stimulation, as seen in allergies, may induce random mutations in rapidly dividing cells, thus elevating cancer risk and promoting stem cell mutations and clonal expansion. These hypotheses offer differing viewpoints on the complex relationship between allergies and cancer (14, 23). However, both of these fundamentally distinct and mutually exclusive theories have been supported by conflicting research findings. Given the complex biological mechanisms underpinning allergic reactions and cancer, along with their widespread implications, both hypotheses may hold true in certain contexts. Furthermore, the impact of allergies is likely to differ depending on the severity of the condition and the location of cancer development (12). Therefore, a prudent approach is necessary when interpreting this matter. IgE is an antibody with potent immune-activating properties and has been shown to play a well-defined role in allergic reactions. IgE has the ability to non-specifically bind to cancer cells, thereby promoting the development of tumor-specific immune memory and serving as an efficacious adjuvant. Simultaneously, it enhances the adhesion of immune cells, including eosinophils, macrophages, and mast cells, which secrete diverse toxic mediators and factors that trigger the lysis of target cells. This mechanism not only aids in anti-tumor activity but also bolsters the recruitment, surveillance, and overall anti-tumor capabilities of immune cells (24, 25). The observed inverse association between allergic diseases and lung cancer risk may be underpinned by enhanced IgE-mediated immune surveillance. Elevated IgE levels characteristic of allergic sensitization can bind FcεRI receptors on effector cells, triggering cytotoxic granule release, pro-apoptotic cytokines, and enhanced tumor antigen presentation, collectively promoting malignant cell clearance and durable anti-tumor immunity (26). IgE enhances the immune system’s capacity to recognize and eliminate tumor cells by activating effector cells such as monocytes, macrophages, and eosinophils. Additionally, IgE promotes antigen presentation through interactions with dendritic cells and B cells, thereby activating CD4 + and CD8 + T cells and further amplifying the anti-tumor immune response (27).

The findings of our study did not reveal a statistically significant association between eczema and lung cancer, which aligns with the results of a previous investigation conducted in Canada (3). Conversely, a cohort study from Taiwan indicated an association between eczema and an elevated risk of lung cancer (11). However, this particular study has been subject to controversy due to its omission of confounding variables such as smoking and alcohol consumption, as well as the absence of histological subtypes for lung cancer and lack of data validation (28). Thus, the reliability of its conclusions remains questionable. While eczema is recognized as an inflammatory disease, its chronic inflammatory state appears to exert a lesser influence on lung cancer risk compared to other allergic conditions. Research indicates that the immune dysregulation associated with eczema may differentially impact various cancer types (29), thereby complicating the relationship between eczema and lung cancer. Our analysis reveals a significant negative correlation between AR and the risk of lung cancer. Furthermore, the preventive hypothesis posits that sneezing induced by allergic rhinitis may facilitate the expulsion of potential mutagenic or carcinogenic toxins from the body prior to their ability to induce malignant tumors. This perspective is also supported by existing research (30), which could elucidate one mechanism through which AR serves as a protective factor against lung cancer.

The chronic inflammation hypothesis proposes that inflammation induced by allergic symptoms may increase the risk of cancer at primary allergy sites (26). Furthermore, the Th2 immune skewing hypothesis posits that allergies may induce a Th2-biased immune response, thereby shifting the immune system away from a potential anti-tumor Th1 response and creating an immunosuppressive microenvironment that facilitates cancer development at sites of allergic inflammation (26). Research has demonstrated that chronic inflammation associated with atopic dermatitis, accompanied by a Th2 skewing, significantly increases the risk of skin cancer while concurrently exhibiting a protective effect against lung cancer, colorectal cancer, and gliomas located distally from these primary allergy sites (31, 32). This indicates that chronic inflammation promotes carcinogenesis in areas directly exposed to inflammatory mediators, whereas enhanced immune responses may protect tissues distant from allergy sites from malignancies (27). Therefore, allergic reactions play a dual role in cancer development—both detrimental and beneficial. Investigating the relationship between allergies and cancer uncovers a complexity that goes beyond a mere increase or decrease in cancer risk, with existing hypotheses inadequately addressing this intricacy. As a result, a study has introduced an integrative hypothesis that combines the theories of immune surveillance, prevention, chronic inflammation, and Th2 immune skewing, thereby highlighting the multifaceted nature of their relationship and the role of IgE. Inflammation induced by allergic reactions is frequently associated with a Th2-skewed immune response, which may elevate the risk of cancer at sites of allergic inflammation. Conversely, the heightened immune surveillance and protective mechanisms triggered by allergic reactions and their symptoms may confer anti-cancer effects in areas distant from the primary manifestations of allergic diseases. This dual influence provides insight into the intricate interplay between IgE, its associated immune responses, and cancer (27).

Our study has several limitations. First, we included only 10 studies related to eczema and AR, which precluded further subgroup analyses for other types of allergies. Furthermore, the small sample sizes of two of the included studies may have compromised the accuracy of our results. Thus, additional research with larger sample sizes is necessary to elucidate the relationship between these conditions. Second, our findings predominantly reflect populations from the Americas, limiting generalizability to underrepresented regions. Geographic variations in environmental triggers, genetic backgrounds, and lifestyle confounders may modulate allergy-cancer relationships. Thus, conclusions should be cautiously applied to non-Western populations until further multinational studies validate these associations. Lastly, our findings should be interpreted in the context of potential misclassification bias arising from questionnaire-based allergy diagnoses. Most included studies relied on self-reported questionnaires to assess eczema and AR, which may introduce recall error or diagnostic misclassification.

## Conclusion

Our study suggests that allergic diseases, such as eczema and AR, are associated with a reduced risk of lung cancer; nonetheless, the precise pathophysiological mechanisms responsible for this observed association remain to be elucidated. The results of this study offer valuable insights into the prevention and treatment of lung cancer.

## Data Availability

The original contributions presented in the study are included in the article/Supplementary material, further inquiries can be directed to the corresponding author.
