# Supplementary figures and images for: Correlation between allergic diseases and lung cancer: a systematic review and meta-analysis
Source: Front Med (Lausanne). 2025 Jul 16;12:1560000. doi: 10.3389/fmed.2025.1560000 (PMC12307174; doi:10.3389/fmed.2025.1560000)

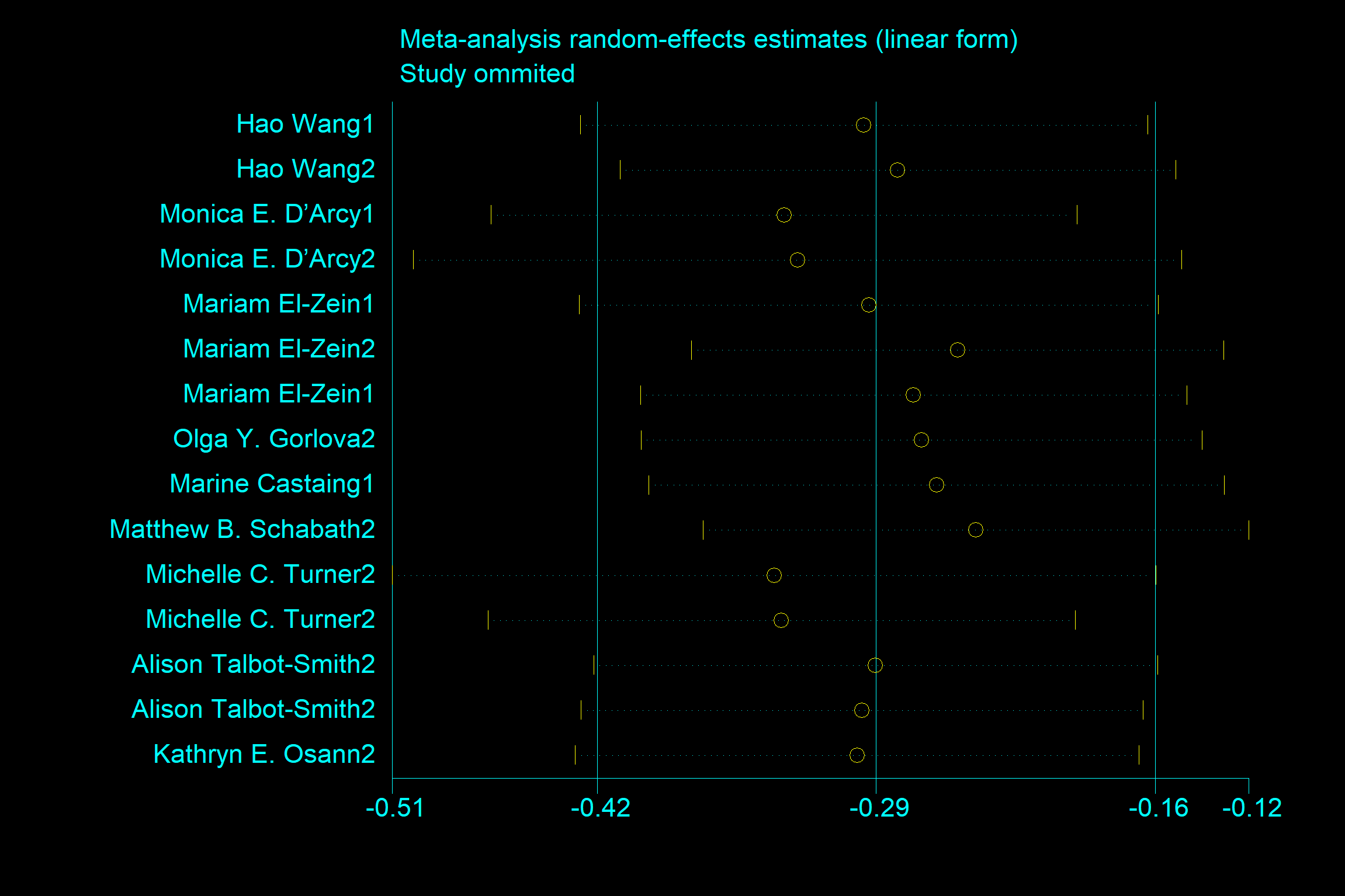

Supplement: Supplementary file 2 [file Image_1.TIF]
