# Supplementary material for: Correlation between allergic diseases and lung cancer: a systematic review and meta-analysis
Source: Front Med (Lausanne). 2025 Jul 16;12:1560000. doi: 10.3389/fmed.2025.1560000 (PMC12307174; doi:10.3389/fmed.2025.1560000)
Supplement: Supplementary file 3 [file Supplementary_file_1.doc]

**Supplementary Material**

PubMed (8/10/2024)

| **Search** | **Query** | **Items found** |
| --- | --- | --- |
| #1 | "Eczema"[MeSH Terms] | 13091 |
| #2 | ((Dermatitis, Eczematous[Title/Abstract]) OR (Eczematous Dermatitis[Title/Abstract])) | 436 |
| #3 | #1OR #2 | 13368 |
| #5 | "Rhinitis, Allergic"[MeSH Terms] | 24521 |
| #6 | ((((Allergic Rhinitides*[Title/Abstract]) OR (Allergic Rhinitis*[Title/Abstract])) OR (Rhinitides,Allergic*[Title/Abstract])) OR (Hay Fever*[Title/Abstract])) | 32293 |
| #7 | #5 OR #6 | 37959 |
| #8 | #3 OR #7 | 50413 |
| #9 | ("Lung Neoplasms"[Mesh]) | 289576 |
| #10 | (((((((((((((((((Lung Neoplasms[Title/Abstract]) OR (Neoplasm, Pulmonary[Title/Abstract])) OR (Pulmonary Neoplasm[Title/Abstract])) OR (Pulmonary Neoplasms[Title/Abstract])) OR (Neoplasms, Lung[Title/Abstract])) OR (Lung Neoplasm[Title/Abstract])) OR (Neoplasm, Lung[Title/Abstract])) OR (Lung Cancer[Title/Abstract])) OR (Cancer, Lung[Title/Abstract])) OR (Cancers, Lung[Title/Abstract])) OR (Lung Cancers[Title/Abstract])) OR (Cancer of Lung[Title/Abstract])) OR (Pulmonary Cancer[Title/Abstract])) OR (Cancer, Pulmonary[Title/Abstract])) OR (Cancers, Pulmonary[Title/Abstract])) OR (Pulmonary Cancers[Title/Abstract])) OR (Cancer of the Lung[Title/Abstract])) | 234793 |
| #11 | #9 OR #10 | 365126 |
| #12 | "Cross-Sectional Studies"[MeSH Terms] OR "cross sectional*"[Title/Abstract] OR "cross-sectional*"[Title/Abstract] OR "Case-Control Studies"[MeSH Terms] OR "case control*"[Title/Abstract] OR "case-control*"[Title/Abstract] OR "Cohort Studies"[MeSH Terms] OR "cohort*"[Title/Abstract] | 3977872 |
| #13 | #8 AND #11 AND #12 | 26 |
